# Do Foreign Investors Affect Carbon Emission Disclosure? Evidence from South Korea

**DOI:** 10.3390/ijerph181910097

**Published:** 2021-09-26

**Authors:** Eunsoo Kim, Suyon Kim, Jaehong Lee

**Affiliations:** 1 Department of Global Business Administration, Sangmyung University, Seoul 03016, Korea; eunsoo1134@gmail.com; 2 Department of Accounting, Jeonbuk National University, Jeonju 54896, Korea; 3 Major in Accounting/Taxation, Division of Business Administration, Kyonggi University, Suwon 16227, Korea; jhong@kyonggi.ac.kr

**Keywords:** voluntary disclosure, carbon emission, Carbon Disclosure Project (CDP), foreign investors, ESG

## Abstract

The purpose of this study is to examine the relationship between foreign investors and voluntary disclosure. Focusing on voluntary disclosure of carbon emissions information and using South Korean firms from 2014 to 2019, we found that foreign investors are likely to voluntarily release information on carbon emissions. Thus, foreign investors play a role in controlling the information gap in a capital market. We also discuss the effect of environmental, social, and governance activities on the relationship between foreign investors and voluntary disclosure. We infer that the analysis result shows that foreign investors motivate firms to improve the environment to prepare for future environmental risks. Our study also suggests solving environmental problems actively, such as responding to climate change, by presenting a direction for policymaking on sustainable management.

## 1. Introduction

This paper examines whether foreign investors affect voluntary disclosure by focusing on carbon emissions. Disclosing information on carbon emissions is vital for corporate sustainability that requires a major innovative transformation of the overall business of allocating and managing resources and energy efficiently [1,2]. Along with the innovative efforts, firms’ responsibility for climate change and ecosystem destruction is inevitable for sustainable growth. According to the World Commission on Environment and Development (WCED), sustainable development is defined as meeting the needs of the present without compromising the ability of generations to meet their own needs. Since then, South Korea has encouraged voluntary participation of firms for its efforts and sustainability, such as enacting laws and regulations to participate in international agreements and the United Nations Framework Convention on climate change. As interests in environmental issues increase, creditors have begun to use related information to make investment decisions. Unlike in the past when investors and creditors focused on generating profits, investors encourage firms to make efforts on environmental responsibilities of reducing carbon emission and disclosing relevant information.

The Carbon Disclosure Project, also known as CDP, is the representative report of showing environmental efforts. CDP began in early 2000 with the support from financial institution investors in Europe, and on behalf of financial institutions, collects and assesses information on carbon emissions, which is a major cause of climate change. Considering the financial institutions agree to provide CDP information, it can be inferred that investors actively use the information of firms’ efforts to reduce carbon emissions.

According to the Korea Exchange, on 13 December 2020, foreign investors flowed into South Korean securities. This figure shows that the total investment inflow into the country reached KRW 212,662 million. South Korea is an emerging market, having experienced two significant outflows of foreign investment [3]. However, as the limitation of foreign investment has gradually relaxed, foreign investors are perceived as more informative and knowledgeable than domestic investors. Furthermore, foreign investors play a significant role in contributing to firms’ overall information environment [4], and their inflows are considered an indicator in the decision-making process of domestic investors [5]. Therefore, management has an incentive to respond to foreign investors, and in this study, we focus on examining whether foreign investors affect voluntary disclosure on carbon emissions provided by CDP.

After examining our hypothesis, we found that the firms with higher foreign investors are likely to disclose environmental information on carbon emissions. We interpret this result that the information gap is reduced when high ratio foreign investors lead to a transparent information environment. Additionally, when the firms are involved in environmental, social, and governance (ESG) activities, this impacts them disclosing information on carbon emissions voluntarily. It is because firms engaged in ESG are more concerned about the environment. According to Heal [6], firms responsible for their environment are more likely to attract foreign investors. It is because they can earn the credibility of their shareholders by disclosing their carbon footprint.

This study adds several contributions to extant prior literature. In the Korean securities market, the influence of foreign investors is continuously increasing, and the ownership ratio is gradually increasing, which provides a research environment where the public pressure of foreign investors can be verified. This study is meaningful in providing evidence on foreign investors’ role in voluntary disclosure using CDP data.

The paper is organized as follows. Section 2 describes theoretical backgrounds and hypotheses. Section 3 explains the research design and sample selection for this study. Then, Section 4 explains the results and discusses them. Finally, Section 5 shows the conclusions.

## 2. Backgrounds and Hypotheses

### 2.1. Voluntary Disclosure of Carbon Emissions

In an efficient market, it is assumed that managers have better information about the firms’ future than outside investors. Therefore, where the accounting regulations are perfectly enforced, management-chosen accounting methods and disclosure can accurately communicate the firm’s business to investors. However, in reality, accounting regulation cannot be thoroughly carried out, so the managers have an option to report either the superior information as it is or adjusted firm performance due to contract cost or political cost.

The content of voluntary disclosure conveys the information not provided by other sources and alleviates information asymmetry. Voluntary disclosure contains more valuable and influential information to investors than mandatory disclosure and earnings announcements [4,7]. Voluntary disclosure can improve stock market liquidity, lower capital costs, and lead to higher corporate value. At the same time, voluntary disclosure creates more transparent information, which leads to increase corporate value. A transparent information environment increases the effectiveness of management oversight and reduces the opportunity to extort private benefits of the management [8].

With the rise of the importance of environmental issues, firms are interested in disclosing environmental information. Among environmental issues, we focus on the information on carbon emissions. Though getting a penalty for carbon emissions, firms choose to disclose carbon emission information because it reduces information asymmetry between the management and outside investors, thus allocating scarce resources efficiently [9]. Firms that disclose accurate information about carbon emissions can convey non-financial information to potential investors for potential future [10,11]. If a firm does not disclose information about carbon emissions, investors would consider it as an adverse signal, which can be harmful to the firm.

The Carbon Disclosure Project (CDP) was established in December 2000 with the support of 35 financial institutions in Europe. Since then, the number of member institutions has steadily increased. As of 2015, the number of CDP-signed financial institutions has reached 822 with assets of USD 95 trillion, and more than 5500 firms from all over the world have reached through CDP. As of 2015, 31 financial institutions have joined as members in South Korea, collecting related information from 250 listed companies. On behalf of financial institutions worldwide, CDP provides accurate information on major carbon emissions, which is a major cause of climate change, and long- and short-term management strategies of companies on related topics from major listed companies around the world. It also provides information that allows the investor to avoid the risk by understanding a specific company’s risk level of climate change in advance.

Each year, firms subject to carbon emissions information voluntarily prepare responses to CDP questionnaires, the content of which is evaluated by a method jointly developed by CDP and PriceWaterhouse Coopers (PWC). The CDP Korea Commission will partially modify the method to suit the Korean situation and evaluate the response’s content for the Korean firms. Then, the evaluation results are published through the CDP official website and the CDP final report.

Disclosing carbon emission information on CDP is not obligatory but optional. The information on carbon emissions disclosed by CDP is reliable based on the following reasons. The market can evaluate the reliability of carbon emission information by comparing a firm’s carbon emission information with other firms belonging to the same industry. The CDP conducts a survey of major firms worldwide, and emissions are legally regulated for the firms belongs to some countries such as the EU. In such cases, the accuracy of carbon emission is ensured, and these firms are subject to comparison with other firms. Therefore, the companies that voluntarily disclose information also use this as a guideline to secure reliability. In addition, although the firms have an option of providing carbon emission to CDP and deciding whether or not to publish the information, once decisions are made to publish in response to the request of CDP, it publishes with continuance [12]. The more times a firm responds to CDP requests, the greater the cost of reporting unreliable or false information in the future. The greater the interests of stakeholders in climate change, the greater the number of reporting firms in the same industry, and the greater the certainty of carbon emission information, the more accurate and reliable information is confirmed by the market. If the firm reports unreliable information in the future, it is known to the market, and the firm can lose credibility in information disclosure other than carbon emissions, which might lead to litigation. Therefore, it can be said that the information reported to the CDP has high reliability even though it is voluntary.

### 2.2. Foreign Investors’ Monitoring

With the liberalization of emerging markets, the behavior of foreign investors has earned considerable attention for the research. Investments of foreign investors have increased steadily after the financial crisis in 1997 in South Korea. As of 10 July 2021, according to the Ministry of Trade, Industry, and Energy, foreign investment in South Korea has increased by 71.5% compared to last year [13].

However, foreign investors are geographically separated from investee firms, have relatively fewer informal channels and language, cultural, and legal barriers. In other words, foreign investors may suffer from an informational gap compared to domestic investors, which leads to incurring costs for the foreign investors to collect information [4]. Therefore, there is a greater incentive for foreign investors to overcome informational opaqueness over informed-domestic investors. Voluntary disclosure is one way to provide information. From the corporate standpoint, firms are motivated to satisfy the foreign investors’ needs, since doing so can attract or retain more foreign investors by expanding their shareholder base. Therefore, reducing information symmetry is one of the foreign investors’ functions. Lee and Cho [14] support that foreign investors are the information intermediaries with superior abilities in analyzing and interpreting, which alleviates information asymmetry, ultimately increasing information transparency [15]. Ahn et al. [16], Chung et al. [17], and Bhojraj and Sengupta [18] suggested that foreign investors are highly skilled groups at collecting and analyzing information. In addition, the roles of foreign investors are supported by the efficient monitoring hypothesis, which postulates that foreign investors are in a superior position of collecting, processing, and trading private information [19,20]. Furthermore, foreign investors are regarded as expert groups with sophisticated research techniques, capital, and experiences from previous transactions compared to domestic investors [21,22]. Additionally, foreign investors are referred to as elite information processors who have critical insight, influencing emerging markets positively.

Foreign investors provide indirect or direct monitoring mechanisms in the emerging markets, and regardless of the form, foreign investors stimulate improvements in corporate governance systems [23]. As Chien [24] and Aggarwal et al. [25] found, foreign investors can motivate firms for improvement by hiring a sufficient number of outside directors. In addition, foreign investors are to have private or superior information since they can acquire private information from the portfolios of their privately hired analysts [26].

That information is used to determine the technological capabilities of a company, a product’s market share, and the intrinsic value of a firm’s earnings forecast. In addition, those privately hired analysts have close acquaintances with the managers. Foreign investors have invested a vast amount of capital [3], and these relationships are used to monitor corporate management decision-making and public policy.

### 2.3. Hypothesis Development

Voluntary disclosure is considered a more significant source than other communicating channels, such as mandatory disclosure and earnings announcements [7]. Additionally, Tsang et al. [4] assert that voluntary disclosure provides value-relevant information to investors that leads to corporate transparency by alleviating information asymmetry among firms.

As the demand for the active response for the continuous and sudden changes in environmental regulations increases, firms are required to disclose environmental issues. As a means to cope with climate changes, CDP is a way to attract the attention of investors. CDP provides firms’ efforts of reducing carbon emission, which the financial institutions agreed on, and disclose its information to the investors that the firms make a great effort in environmental improvement.

In order to secure competitiveness in a market, sustainable corporate activities, such as socially responsible activities, setting international standards, and information disclosure of carbon emissions, are necessary conditions for the international community. It means that while increasing corporate awareness and corporate value, it is possible to induce continuous investment by building trust among stakeholders. While prices, product quality, design, marketing, and services are the factors that determine a firm’s competitiveness in the past, currently, firms’ environmental impact, environmental activities, and environmental performances are essential for the firms’ sustainable existence.

However, there may be direct costs to disclosing environmental information that may incur a decrease in profits. Additionally, it may be argued that excessive concentration may be rather harmful to corporate value, such as conflicting interests between shareholders and management due to increased corporate costs. At the same time, the profitable business proposals and investment strategies will be alienated in order to raise interest in environmental management.

Outside investors can obtain information on whether the firms fulfill their environmentally responsible activities by relying solely on the information disclosed by the firm. Inferiority to the information can be severe for foreign investors, but foreign investors will overcome the barrier. To overcome the geographic disadvantages and reduce the information gap, foreign investors will demand information on firms’ environmental activities [4]. Foreign investors want to invest in firms with transparent corporate disclosure to solve their information imbalances [27]. Additionally, foreign investors are more interested in demanding voluntary disclosure since they have the background of a well-established disclosing environment [28]. At the same time, foreign investors play a role as a monitoring system, promoting the improvement of the firm [23]. Foreign investors can suggest that management hire a sufficient number of outside directors to enhance the firms’ transparent accounting environment. Therefore, we postulate that foreign investors are likely to demand voluntary disclosure on carbon emissions, and we set up the first hypothesis as follows.

**Hypothesis** **1.** *Firms with a high ratio of foreign investors are likely to disclose information on carbon emissions voluntarily.*

Foreign investors invest in highly transparent companies that they are familiar with because their information is inferior to domestic investors [29,30]. Investing in the firms engaged in ESG is one way to solve the problem. ESG is a significant standard in the international community for measuring sustainable development by expanding and strengthening the concepts of green economy, corporate social responsibility, and environmentally responsible investment [31]. In South Korea, the Korea Corporate Governance Service (KCGS) provides the ESG scores and encourages firms to involve in socially responsible practices. Firms participating in environmental initiatives signal that they are responsible corporate citizens with greater transparency and less misreporting [32,33]. In addition, from the point of stakeholder value creation theory, firms that invest in ESG strengthen reputation, thereby attracting more investment [34,35]. Additionally, ESG investments can suppress the motivation of private earnings management [36]. Therefore, stakeholder value creation theory suggests that investing in ESG is viewed as more responsible. Though foreign investors unavoidably come from overseas, they are superior in collecting, processing, and analyzing data. At the same time, foreign investors with the background of better disclosure standards request for more disclosure [37]. Therefore, foreign investors choose to invest in firms that are engaged in ESG and we set up a hypothesis connecting to voluntary disclosure.

**Hypothesis** **2.** *Foreign investors in the firms engaged in ESG are likely to disclose information on carbon emissions voluntarily.*

## 3. Research Design and Sample Description

### 3.1. Research Model

Hypothesis 1 predicts whether foreign investors affect managerial voluntary disclosure of carbon emissions. In order to investigate the role of foreign investors on voluntary disclosures, the following logistic regression model is employed to test the first hypothesis.

(1) $$\begin{array}{c} {\mathrm{Voluntary}}_{t}=\alpha_{0}\;+\beta_{1}{\mathrm{For}}_{t}+\beta_{2}{\mathrm{Size}}_{t}+\beta_{3}{\mathrm{Lev}}_{t}+\beta_{4}{\mathrm{Roa}}_{t}\\ +\beta_{5}{\mathrm{Growth}}_{t}+\beta_{6}{\mathrm{Loss}}_{t}+\beta_{7}{\mathrm{Da}}_{t}+\beta_{8}{\mathrm{Vol}}_{t}+\mathrm{Ind}+\mathrm{Yr} \end{array}$$

The definitions of the independent, dependent, and control variables used in this study are described in Table 1.

In Equation (1), Voluntary measures whether the firms voluntarily disclose information on carbon emissions obtained from the Carbon Disclosure Project (CDP). The control variables are Size, Lev, Roa, Growth, Loss, Da, Vol, Ind, and Yr. Size is a proxy for firms’ information environment [39]. Large-sized firms disclose information about themselves, and that information is used by the stakeholders. Stanny and Ely [12] and Stanny [40] report that firms response CDP are large-sized. Thus, the firm size is included in the model. Lev is a measurement of the debt ratio. Sengupta [41] asserted that high-quality disclosure tends to have lower debt costs. Therefore, in the case of a firm with a high debt ratio, the Lev variable may have a positive relationship because it has an incentive to disclose good quality information to reduce the cost of debt. CDP-signed financial institutions use CDP reports to obtain the firm’s environmental information and use it when making investment decisions. Therefore, these firms with high liabilities are more likely to disclose carbon emissions in response to the CDP survey. Roa is a variable that expresses the firm’s profitability. According to the economic theory, firms with good performance are incentivized to separate themselves from bad performers [42]. Thus, we expect that the firms with high profits are inclined to disclose environmental information. Growth is included in the model since foreign investors are likely to invest in a firm with the potential to grow [28]. As Jung [43] suggested, firms that predicted an increase in profits tend to disclose management forecast information voluntarily. Simnett et al. [44] reported that the firms that make sustainable investments, such as in renewable energy, are more likely to receive positive reviews from stakeholders. These positive evaluations are likely to lead to profits and are expected to increase the firm’s sales. Thus, we include firms’ growth in our model. Loss is an indicator variable that is coded as 1 if the firm is in financial difficulty. Firms that are in financial difficulty are less credible in voluntary disclosure [45]. Financially troubled firms are more likely to avoid disclosing sensitive information to avoid undermining their credibility. However, in the case of financially superior firms, there are incentives to provide accurate information disclosure to maintain credibility. The relative price change determines the volatility of a firm’s performance for 360 calendar days. This measure, known as Vol, is often used to evaluate the firm’s performance or uncertainty in the business environment [46]. The more uncertain the business environment, the greater the information asymmetry between managers and outside investors [47], but it is reported that voluntarily disclosing information alleviates information asymmetry [48]. However, if the volatility of firm performance includes the information that managers cannot obtain it in advance, the firm’s volatility negatively impacts the information disclosure [49]. Da is a calculation of discretionary accruals by Kothari et al. [38]. There is an information gap between management and stakeholders. Managers have superiority in accessing to a private information when making decisions. However, since earnings management adversely affects firm value, managers have an incentive to manage earnings to an acceptable level [50]. At the same time, firms involved in sustainable management are more likely to have a good reputation that helps reduce information asymmetry [51]. Disclosing information on carbon emission is recognized as a trait of firms that are environmentally concerned, resulting in a positive reputation. Therefore, it is expected that firms that manage earnings are expected to offset the negative impact by disclosing carbon emission information. We estimate Da in accordance with Kothari et al. [38] described in Equation (2). The regression coefficient of Equation (2) is estimated by the industry year for the firms to be analyzed. The period of time is from 2014 to 2019, and the Korean Standard Industrial Classification Code is used for industry.

(2) $${\mathrm{Ta}}_{t}/A_{t}=\alpha_{0}+\beta_{1}(1/A_{t})+\beta_{2}(△\mathrm{Sales}\;-\;△\mathrm{Ar})/A_{t}+\beta_{3}{\mathrm{Ppe}}_{t}\;/A_{t}+\beta_{4}{\mathrm{Roa}}_{t}+\varepsilon_{t}$$

where, Ta = Net income − cash flow from operation; A = Total assets; Sale = Sales revenue; Ar = Accounts receivables; Ppe = Plant, property, and equipment; Roa = Net income/total assets.

### 3.2. Data Selection

Table 2 shows the data selection process. First, firms listed in the Korea Stock Exchange (KSE) and the Korea Securities Dealers Automated Quotation (KOSDAQ) are selected from 2014 to 2019. The ESG scores are privileged data provided by KCGS. Then, firms in the financial industry and without financial data are deleted. Therefore, the final sample is 1760. The top and bottom one percent of all variables are winsorized to alleviate the outlier effect.

## 4. Empirical Results

### 4.1. Descriptive Statistics

Table 3 shows the descriptive statistics of the main variables. The mean value of voluntary is 0.268, implying that 26% of firms in the sample voluntarily disclose information on carbon emissions requested by CDP. It appears that about 26.8% of the sample firms that received surveys requested through CDP voluntarily disclose information. The mean value of shares held by foreign investors is 0.069. The maximum value, not tabulated, is 0.897, implying that the foreign shareholding ratio that holds the largest foreign share accounted for about 89.7% of the total shares.

Table 4 shows the correlation of the main variables. The correlation between voluntary and foreign is positive, implying that as foreign investors increase, the firms are likely to disclose information on carbon emissions. Additionally, a positive correlation between ESG and foreign investors is confirmed. Chaebol-affiliated firms (Chaebol) are positively correlated with voluntary disclosure on carbon emissions. The firm with information asymmetry is negatively correlated with voluntary disclosure.

### 4.2. Regression Results and Discussion

This section presents the results of a logistic analysis that considers the firms’ characteristic variables that influence voluntary disclosure. Table 5 describes the result of the relationship between foreign investors and voluntary disclosure of carbon emissions. The coefficient of foreign is positive at 5% level, after controlling the firm environment. The result supports the first hypothesis, implying that firms with a high foreign investor ratio are likely to disclose environmental information about carbon emissions voluntarily. Foreign investors tend to focus on corporate value and transparency, and actively monitor the opportunistic management decisions, thereby mitigating the agency problem [52]. In addition, foreign investors demand to monitor and supervise functions, decrease the likelihood of reporting material weaknesses in the internal control system, and ultimately lead to transparency in firms’ information.

Therefore, our result aligns with the prior research that foreign investors require better disclosure standards and demand greater disclosure to reduce the information gap [53]. In the study of Jeon [54], it is reported that foreign investors would entice management to provide reliable accounting information. Additionally, Oh and Sohn [55] reported increased fair disclosure due to increasing demand for transparency of firms with high equity interests of foreign shareholders. Collectively, foreign investors are likely to disclose information on the environment, which is considered part of sustainable management [54].

Table 6 documents the result of the ESG effect on the relationship between foreign investors and voluntary disclosure. The coefficient of the interaction term, For X ESG, is 0.023 and significantly positive. It means that the foreign investors in the firms engaged in ESG are likely to disclose information on carbon emissions voluntarily.

Foreign investors have geographical disadvantages in that they are not familiar with the local situation. To overcome this risky situation, foreign investors choose to invest in reliable and legitimate firms acting as responsible firms [56]. As Heal [6] argued, corporate socially responsible activities can reduce stakeholder conflicts and maximize stakeholder credibility. Additionally, foreign investors tend to choose firms that voluntarily disclose information, including non-financial information such as ESG, which prolongs CSR that encompasses environmental, social, and corporate governance. The result suggests that in the transparent information environment, foreign investors cannot disclose environmental information voluntarily.

### 4.3. Additional Analysis

Agency problems occur in firms from developed countries due to the separation of ownership and management. A pyramidal structure is a type of ownership structure that occurs when a group of companies controls all of its member firms. It is called Chaebol in South Korea, which indicates an affiliate of a large business group designated by the Korea Fair Trade Commission (KFTC). Chaebols have grown with extensive government support as a part of the economic recovery efforts and account for the majority of the market capitalization of the securities market. Controlling shareholders’ value of Chaebol is positively associated with the long-term value of the firm rather than short-term. For example, under Chaebol ownership, the value of Tobin’s Q, which represents the long-term value, is high, whereas Earnings Before Interest, Tax, Depreciation, and Amortization (EBITDA), which indicates short-term firm performance, decreases [57]. It can be inferred that management decision-making is guided in the direction of increasing corporate value from a long-term perspective rather than short-term corporate performance. Kang et al. [58] assert that managers are reluctant to invest in Research and Development (R&D) investment, which leads to lower EBITDA, even though R&D investments increase the value of a firm from a long-term perspective.

Table 7 shows the results of the logistic analysis on the impact of Chaebol. The estimated coefficient of interaction term of For X Chaebol is significantly positive at the 1% level. The result is consistent with the main finding that foreign investors’ monitoring mechanism is evident for Chaebol firms. Since foreign investors are from overseas, they are likely to choose effective corporate governance [59]. In other words, foreign investors rely more on effective corporate governance, which limits management and controlling shareholders from exacerbating shareholders’ interest. Na et al. [60] suggest that a high proportion of foreign investors positively impacts the goal of responding to climate change. Firms’ environmental efforts result in positive evaluations by foreign investors, and those efforts affect the disclosure of environmental information [54].

Foreign investors’ preferences may vary depending on the level of the agency problem between management and shareholders. Mostly, investors are more likely to favor firms with strong management teams and favorable financial conditions. Due to information asymmetry, a manager may benefit from the private information available to them. It is widely believed that rent-seeking managers are incentivized to select projects that will cover up their firm’s performance [3]. We use stock return volatility to test the influence of information asymmetry between the managers and external investors [61]. The level of information asymmetry is measured as standard deviation of market excess returns per week throughout a year. Weekly returns are computed from Thursday to Wednesday to minimize the effect of bid–ask bounce volatility. High stock volatility is an indicator that shows how firms are high in information asymmetry.

Table 8 shows the result of the association between foreign investors and voluntary disclosure depending on the level of information asymmetry. As shown in Table 8, the coefficient of For X Ia is positive and significant, implying that foreign investors prefer disclosing environmental information is evident in high information asymmetry environments. As Kim and Verrechia [62] and Khanna and Palepu [63] suggest, foreign investors are superb at processing information and providing enhanced monitoring. Thus, in a market with high information asymmetry, the fact that foreign investors act as a monitoring mechanism is evident.

## 5. Conclusions

Climate change is a risk factor that threatens all humankind. Many countries have already experienced enormous damage due to extreme weather events caused by climate change, which has a great impact on business activities. Climate change is recognized as an important factor not only in the ecosystems and human living environment but also in economic growth. The firm’s response to climate changes goes beyond the choice, and now it is a reflection of consumer needs and corporate obligation. The major cause of climate change is carbon emissions. In the field of accounting area, where making a high profit is key to survive in a capital market, controlling for carbon emissions is vital for the firms’ sustainability.

This study examines targets of the firms listed on the securities market in South Korea from 2014 to 2019 and conducted a test on the role of foreign investors, who can exert an important influence on corporate management decision-making and voluntary disclosure, focusing on disclosing information on carbon emissions. Voluntarily disclosing the information is at managerial discretion and a valuable channel to communicate with outside. The result suggests that the information gap is reduced when many foreign investors yield a transparent information environment. At the same time, foreign investors play an important role as a control device in management decision-making. These results suggest that the foreign investors monitor management decision-making, enabling transparent management and influencing voluntary disclosure positively. We also conduct our analysis incorporating firms’ ESG. Our result shows that foreign investors who choose firms engaged in ESG activities are likely to disclose information on carbon emissions. In other words, foreign investors tend to choose transparent firms with superior abilities. Moreover, ESG participants are considered valuable mechanisms for gaining trust and reputation internationally [56]. Thus, firms with ESG and a high ratio of foreign investors are likely to disclose information on carbon emissions. We also conducted an additional analysis of Chaebol. A Chaebol is a unique characteristic of corporate governance in South Korea, and the results suggest that foreign investors in Chaebol firms are likely to disclose information on carbon emissions. Additionally, in firms with high information asymmetry, foreign investors monitor and choose to disclose information on carbon emissions. Collectively, foreign investors have an impact on sustainable management, such as corporate environmental improvement efforts. In such a change, if a firm recognizes the risk of climate change and actively participates in overcoming it, it can be an opportunity rather than a limitation.

Our study contributes to the extant literature. First, it has the practical implication that foreign investors are interested in not only financial information, but also non-financial information, which provides a direction for policy making of sustainable management. Foreign investors are a superior group at dealing with information, and they prefer firms with high profits. As foreign investors make investments based on accounting information in the past, non-financial information is vital when they invest.

The result shows that foreign investors play a role in controlling the opportunistic behavior of management, which can mislead regulators and shareholders to ensure the credibility of the firm’s disclosure. The results show that high-level foreign investors play an important role in promoting reliable voluntary disclosure and reducing information asymmetry between investors and management. It also provides policy implications such as regulating relevant supervisors on listed companies with a relatively low level of foreign investors.

Our study is subject to limitations. For example, although foreign investors are powerful in emerging markets such as South Korea, it might generate different results in countries with different capital environments. Additionally, if the CDP data over time accumulates, future research can confirm the results.

## Figures and Tables

**Table 1:** Variable definition.

Voluntary	If firms voluntarily disclose the information of carbon emission is equal to 1, and 0 otherwise
For	Percentage of shares held by foreign investors
ESG	Natural logarithm of scores of ESG
Chaebol	Chaebol-affiliated firms
Ia	Information asymmetry
Size	log(total asset)
Lev	Total liabilities/total assets
Roa	Net income/total assets
Growth	(Sales in current year—sales in prior year)/sales in the prior year
Loss	Equal to 1 if the firm reports losses, 0 otherwise
Da	Discretionary accruals measured by the model in Kothari et al. [38]
Vol	The average trading volume of firm
Ind	Industry dummies
Yr	Year dummies

**Table 2:** The data selection process.

CDP surveyed firms with December year-end in years 2014–2019	2408
Less:	
No ESG data	390
No financial data, financial institution	258
Final observation	1760

**Table 3:** Descriptive statistics.

Variables	Mean	STD	Q1	Median	Q3
Voluntary	0.268	0.443	0.000	0.000	1.000
For	0.069	0.119	0.005	0.019	0.076
ESG	3.212	0.585	2.833	3.218	3.555
Chaebol	0.090	0.286	0.000	0.000	0.000
Ia	0.650	0.477	0.000	1.000	1.000
Size	25.387	1.429	24.444	25.178	26.075
Lev	0.400	0.209	0.230	0.393	0.549
Roa	−0.053	0.436	−0.035	0.042	0.100
Growth	0.028	0.331	−0.090	0.035	0.158
Loss	0.306	0.461	0.000	0.000	1.000
Da	0.000	0.124	−0.045	0.007	0.055
Vol	0.031	0.012	0.020	0.030	0.040

Notes: See Table 1 for definitions of the variables.

**Table 4:** Pearson correlation.

	(1)	(2)	(3)	(4)	(5)	(6)	(7)	(8)	(9)	(10)	(11)	(12)
(1) Voluntary	1.00	0.28	0.43	0.29	−0.06	0.45	0.18	−0.02	−0.05	0.04	−0.04	−0.17
		<0.0001	<0.0001	<0.0001	0.01	<0.0001	<0.0001	0.44	0.04	0.09	0.09	<0.0001
(2) For		1.00	0.33	0.22	−0.09	0.54	−0.09	0.12	0.02	−0.16	0.00	−0.21
			<0.0001	<0.0001	<0.0001	<0.0001	<0.0001	<0.0001	0.00	<0.0001	0.69	<0.0001
(3) ESG			1.00	0.40	−0.04	0.50	0.16	0.09	−0.02	−0.11	−0.02	−0.22
				<0.0001	0.00	<0.0001	<0.0001	<0.0001	0.21	<0.0001	0.24	<0.0001
(4) Chaebol				1.00	−0.04	0.42	0.11	0.05	0.02	−0.06	0.00	−0.12
					<0.0001	<0.0001	<0.0001	<0.0001	0.01	<0.0001	0.75	<0.0001
(5) Ia					1.00	0.06	−0.01	0.05	0.03	0.03	0.04	0.41
						<0.0001	0.04	<0.0001	<0.0001	0.00	<0.0001	<0.0001
(6) Size						1.00	−0.11	0.22	0.09	−0.24	0.05	−0.24
							<0.0001	<0.0001	<0.0001	<0.0001	<0.0001	<0.0001
(7) Lev							1.00	−0.31	−0.01	0.27	−0.21	0.17
								<0.0001	0.14	<0.0001	<0.0001	<0.0001
(8) Roa								1.00	0.20	−0.51	0.48	−0.23
									<0.0001	<0.0001	<0.0001	<0.0001
(9) Growth									1.00	−0.24	0.14	0.01
										<0.0001	<0.0001	0.13
(10) Loss										1.00	−0.37	0.26
											<0.0001	<0.0001
(11) Da											1.00	−0.06
												<0.0001
(12) Vol												1.00

(1) See Table 1 for definitions of the variables.

**Table 5:** The relationship between foreign investors and voluntary disclosure.

Variables	Est.	Wald x^2^
Intercept	−30.125	238.104 ***
For	0.009	4.366 **
Size	1.020	219.067 ***
Lev	0.164	14.249 ***
Roa	−2.079	4.209
Growth	−0.232	1.702
Loss	0.156	0.550
Da	0.205	0.078
Vol	−8.595	1.600
Ind	Included	
Yr	Included	
Pseudo R^2^	0.29	
Log Likelihood	532.35 ***	
Observations	1760	

(1) *, **, and *** indicate significance at the 10%, 5%, and 1% levels, respectively. (2) See Table 1 for definitions of the variables.

**Table 6:** The effect of ESG on the relationship between foreign investors and voluntary disclosure.

Variables	Est.	Wald x^2^
Intercept	−27.6756	72.1795 ***
For	−0.085	2.590
ESG	0.764	4.811 **
For X ESG	0.023	2.834 *
Size	0.807	54.704 ***
Lev	3.260	38.763 ***
Roa	−0.036	0.006
Growth	−0.299	0.714
Loss	0.106	0.176
Da	0.020	0.001
Vol	−39.483	8.212 ***
Ind	Included	
Yr	Included	
Pseudo R^2^	0.31	
Log Likelihood	372.36 ***	
Observations	1760	

(1) *, **, and *** indicate significance at the 10%, 5%, and 1% levels, respectively. (2) See Table 1 for definitions of the variables.

**Table 7:** The effect of Chaebol-affiliated firms on the relationship between foreign investors and voluntary disclosure.

Variables	Est.	Wald x^2^
Intercept	−24.026	181.090 ***
For	0.413	2.740 *
Chaebol	0.659	20.341 ***
For X Chaebol	1.421	9.673 ***
Size	0.783	150.949 ***
Lev	0.083	3.142 *
Roa	0.357	2.757 *
Growth	−0.023	0.012
Loss	−0.105	0.320
Da	−1.315	2.189
Vol	−21.866	15.367 ***
Ind	Included	
Yr	Included	
Pseudo R^2^	0.275	
Log Likelihood	568.18 ***	
Observations	1760	

(1) *, **, and *** indicate significance at the 10%, 5%, and 1% levels, respectively. (2) See Table 1 for definitions of the variables.

**Table 8:** The effect of information asymmetry on the relationship between foreign investors and voluntary disclosure.

Variables	Est.	Wald x^2^
Intercept	−26.800	254.306 ***
For	0.196	0.345
Ia	−0.551	1.713
For X Ia	0.861	3.874 **
Size	0.909	232.910 ***
Lev	0.076	2.441
Roa	0.360	2.556
Growth	0.001	0.000
Loss	−0.195	1.051
Da	0.407	0.349
Vol	−24.053	17.376 ***
Ind	Included	
Yr	Included	
Pseudo R^2^	0.262	
Log Likelihood	537.57 ***	
Observations	1760	

(1) *, **, and *** indicate significance at the 10%, 5%, and 1% levels, respectively. (2) See Table 1 for definitions of the variables.

## Data Availability

Not applicable.
